# A xCT role in tumour-associated ferroptosis shed light on novel therapeutic options

**DOI:** 10.37349/etat.2022.00101

**Published:** 2022-10-25

**Authors:** Daniela Criscuolo, Francesco Morra, Angela Celetti

**Affiliations:** 1 Institute for the Experimental Endocrinology and Oncology, Research National Council, CNR, 80131 Naples, Italy; 2 Department of Molecular Medicine and Medical Biotechnology, University of Naples Federico II, 80131 Naples, Italy; 3 Department of Medicine and Surgery, University of Milano-Bicocca, 20900 Monza, Italy; National University of Singapore, Singapore

**Keywords:** Solute carrier family 7 member 11 (SLC7A11), reactive oxygen species (ROS) tolerance, ferroptosis-regulators, coiled-coil domain containing 6 (CCDC6), cancer therapy

## Abstract

Solute carrier family 7 member 11 (SLC7A11; also known as xCT), a key component of the cystine/glutamate antiporter, is essential for the maintenance of cellular redox status and the regulation of tumor-associated ferroptosis. Accumulating evidence has demonstrated that xCT overexpression, resulting from different oncogenic and tumor suppressor signaling, promotes tumor progression and multidrug resistance partially via suppressing ferroptosis. In addition, recent studies have highlighted the role of xCT in regulating the metabolic flexibility in cancer cells. In this review, the xCT activities in intracellular redox balance and in ferroptotic cell death have been summarized. Moreover, the role of xCT in promoting tumor development, drug resistance, and nutrient dependency in cancer cells has been explored. Finally, different therapeutic strategies, xCT-based, for anti-cancer treatments have been discussed.

## Introduction

Solute carrier family 7 member 11 (SLC7A11), also known as xCT, is the functional light chain subunit of the system x_c_^−^, an amino acid antiporter that mediates the uptake of cystine in exchange for glutamate [1]. xCT has a key role in maintaining the intracellular redox balance by promoting the synthesis of reduced glutathione (GSH), the primary antioxidant molecule, for which cystine is the rate-limiting precursor [2]. Moreover, xCT is a critical regulator of ferroptosis, a regulated form of cell death driven by oxidative damage to membrane lipids [3]. As well as apoptosis, ferroptosis induces the elimination of damaged or oxidatively stressed cells by acting with a tumor suppressive mechanism [4]. Consistently, oncogenes activation and/or tumor suppressor loss cases may allow cancer cells to bypass ferroptosis through the induction of xCT expression [5]. Interestingly, xCT overexpression has been observed in different human cancers and is associated with poor prognosis and multidrug resistance [6]. Noteworthy, recent studies shed light on the emerging role of xCT in inducing nutrient dependency on cancer cells, paving the way for alternative metabolism-based strategies to induce ferroptosis [5]. In this review the xCT role in protecting cancer cells from oxidative stress and ferroptosis will be discussed, highlighting the importance of xCT-mediated ferroptosis inhibition in cancer development and multidrug resistance. Furthermore, we will explain the metabolic consequences of xCT overexpression. Lastly, we will examine the potential of the xCT targeting to treat cancer and to overcome the drug resistance.

## The function of xCT in regulating oxidative stress and ferroptotic responses

Reactive oxygen species (ROS) are highly reactive chemicals. They are mainly generated as by-products of aerobic metabolism and are involved in several cellular processes such as regulators of different signaling pathways [7]. The imbalance between ROS production and the reactive product removal by the cellular antioxidant systems may result in oxidative stress which exerts a dual role in cancer development. In fact, moderate ROS levels sustain tumor initiation and progression by promoting cancer cell proliferation, survival, angiogenesis, migration, and drug resistance [8]. Differently, excessive ROS levels can induce cancer cell death [8]. Cancer cells generally display high ROS levels counterbalanced by an increase in antioxidant defense systems [9]. Therefore, tight regulation of intracellular redox balance through pro-oxidant and anti-oxidant programs is crucial for cancer cell proliferation and survival.

The cystine/glutamate antiporter system xCT plays a key role in maintaining cellular redox status by promoting the uptake of cystine, the oxidized dimeric form of cysteine present in the oxidizing extracellular environment [1]. Due to the reducing intracellular milieu, imported cystine is rapidly converted to cysteine, a proteinogenic amino acid that serves as a rate limiting-precursor of GSH, the major antioxidant molecule [2].

GSH is a tripeptide composed of glycine, glutamate, and cysteine. The GSH biosynthesis involves two ATP-consuming reactions [10]. In the first reaction, the enzyme γ-glutamate cysteine ligase (γ-GCS) catalyzes the conjugation of cysteine with glutamate. Then, the GSH synthase (GS) mediates the addiction of glycine to the dipeptide γ-glutamyl-cysteine forming GSH. The antioxidant action of GSH depends on its ability to reduce ROS in a reaction catalyzed by different GSH peroxidases (GPXs). In this process, GSH is oxidized to GSH disulfide (GSSG), which in turn is reduced and recycled to GSH by GSH reductase (GR) at the expense of nicotinamide adenine dinucleotide phosphate (NADPH). Moreover, GSH operates the detoxification of reactive electrophiles including xenobiotics and secondary metabolites formed during cell metabolism. In particular, electrophile compounds form conjugates with GSH, spontaneously or enzymatically due to the action of the GSH S-transferases (GSTs). The GSH adducts are then exported from the cell by multi-drug resistance proteins [11]. Besides contributing to GSH synthesis, cysteine can generate additional molecules with antioxidant properties including cysteine persulfide, hydrogen sulfide, and taurine [12].

Redox imbalance towards pro-oxidant conditions can induce a regulated form of oxidative cell death called ferroptosis is driven by the lethal accumulation of lipid hydroperoxides that induces cell membrane damage [13]. The term ferroptosis was conceived in 2012 to describe a non-apoptotic cell death induced by inhibition of cystine uptake [3]. However, earlier studies identified cystine as an essential component for the proliferation of cells in culture. Moreover, the absence of cystine in the culture medium resulted in cell death, upon GSH depletion, which could be prevented by treatment with lipophilic antioxidants [14, 15]. The oxidation of polyunsaturated fatty acids in membrane phospholipids, the presence of redox-active iron responsible for the oxidation of membrane polyunsaturated fatty acids and the loss of repair systems that eliminate lipid hydroperoxides represent the characteristic hallmarks of ferroptosis [16]. Increased intracellular ROS levels are essential to trigger the crucial event for ferroptosis, the oxidative damage of lipid membranes [17]. The main cellular sources of ROS involved in this process are iron metabolism, the mitochondrial respiratory chain and the activity of NADPH oxidases (NOXs) [18]. Excess of free redox-active iron (Fe^2+^) can directly generate ROS through the Fenton reaction by producing hydroxyl radicals (•OH) that promote lipid peroxidation [3]. Therefore, the regulation of proteins involved in iron metabolism, such as ferritin and transferrin receptors, affects ferroptosis sensitivity in cancer cells [19]. Generation of mitochondrial ROS mainly occurs during oxidative phosphorylation at the electron transport chain [20]. Mitochondrial ROS play a crucial role in ferroptotic cell death [21]. Indeed, inhibition of respiratory complexes suppresses lipid ROS accumulation and ferroptosis induced by cystine deprivation [21]. Moreover, the rewiring of cancer cell metabolism towards oxidative phosphorylation through genetic disruption of glycolysis increases oxidative stress and ferroptosis sensitivity [22]. Another important source of ROS is the NOX protein family, which are membrane-associated proteins that transfer electron across biological membranes [23]. ROS derived from NOXs have been shown to promote ferroptosis by inducing lipid peroxidation while NOX inhibition significantly reduces erastin-induced ferroptosis [3, 24]. Notably, a large-scale testing of small molecules in 60 human cell lines revealed that NADPH abundance can predict sensitivity to ferroptosis-inducing compounds [25].

The inhibition of xCT/GSH/GPX4 axis has been recognized as one of the main drivers of ferroptosis [13]. Mechanistically, depletion of GSH impairs the activity of GPX4 which, by using GSH as a cofactor, is able to reduce toxic lipid hydroperoxides to corresponding non-toxic alcohols, leading to massive lipid peroxidation and cell death [13, 26]. Consistently, while xCT overexpression protects cancer cells from oxidative stress and ferroptotic cell death [3, 27], the genetic ablation or pharmacological inhibition of xCT can induce ferroptosis.

## xCT-mediated ferroptosis inhibition contributes to tumor development

Resistance to cell death is one of the cancer hallmarks [28]. It is well established that defects in the apoptotic pathway contribute to the expansion of the neoplastic cell population, induce the escape of cancer cells from immune surveillance, and reduce the efficacy of anticancer therapies [29]. Emerging evidence suggests that ferroptosis, similar to apoptosis, exerts a tumor suppressive function by removing cells exposed to metabolic stress conditions [4]. In line with this view, xCT has been found overexpressed in several cancer types, including non-small cell lung cancer (NSLCL), triple-negative breast cancer (TNBC), liver carcinoma, glioma, and renal cell carcinoma, and its elevated expression often correlates with poor prognosis and drug resistance [30–34].

Moreover, several studies have demonstrated that activation of proto-oncogenes, as well as loss of tumor suppressors, enhance the expression of xCT that promotes tumor development by mediating ferroptosis suppression [5, 6]. The rat sarcoma virus (RAS) family genes (*HRAS*, *KRAS*, *NRAS*) are the most frequently mutated proto-oncogenes in human cancer [35]. RAS genes encode for small guanosine triphosphate (GTP)-binding proteins essential to transmit signals from plasma membrane receptors to various intracellular signaling cascades which control cell proliferation, survival, migration, differentiation, and gene expression [36]. Abnormal activation of RAS proteins significantly contributes to several aspects of the malignant phenotype, including uncontrolled proliferation, apoptosis escape, invasiveness, and the ability to induce new blood vessel formation [37]. RAS-mediated malignant transformation is mainly attributed to the induction of the pro-proliferative rapidly accelerated fibrosarcoma (RAF)/mitogen-activated protein kinase kinase (MEK)/extracellular signal-regulated kinase (ERK) pathway and anti-apoptotic phosphatidylinositol 3-kinase (PI3K)/protein kinase B (AKT) signaling [37]. In addition, control of redox balance has recently been proposed to support RAS oncogenic functions [38, 39]. However, the exact role of ROS in RAS-induced transformation and tumor growth is still a controversial issue as RAS can activate pro-oxidant and anti-oxidant programs which both may contribute to tumor development [40]. Several studies have demonstrated that activation of KRAS, the predominantly RAS mutated isoform, increases intracellular ROS production. The activation of ROS-producing enzymes, such as NADPH oxidase and cyclooxygenase-2, and the increase of mitochondrial metabolism are described as the main pro-oxidant mechanisms activated by KRAS [41–43]. The generation of ROS contributes to KRAS-mediated transformation by inducing cell proliferation and genetic instability [44]. On the other hand, oncogenic KRAS induces the expression of xCT determining an increase in cystine uptake and GSH biosynthesis [39, 45]. Notably, inhibition of xCT selectively kills KRAS-mutant cancer cells *in vitro* and strongly attenuates oncogenic RAS transformation in xenograft models, highlighting an unappreciated function of the antioxidant program to RAS-driven tumourigenesis [39, 45]. Mechanistically, KRAS controls xCT expression via the transcription factor E26 transformation-specific (ETS-1) which, in synergy with activating transcription factor 4 (ATF4), transactivates the xCT promoter downstream of the RAF-MEK-ERK signaling cascade [45]. Moreover, it has been reported that oncogenic KRAS can promote xCT transcription also through nuclear factor E2-related factor 2 (NRF2), the master regulator of oxidative response [39].

Interestingly, the tumor suppressor genes *p53*, BRCA1-associated protein 1 (*BAP1*), and coiled-coil domain containing 6 (*CCDC6*) have been reported to regulate ferroptosis by modulating the xCT expression. *p53* is the most frequently mutated gene in human cancers [46]. Traditionally, the tumor-suppressive role of p53 has been attributed to its ability to induce cell cycle arrest, senescence, and apoptosis. However, several studies have revealed that regulation of cellular metabolism and maintenance of redox balance contribute to p53-mediated tumor suppression [27]. Indeed, p53 has been shown to inhibit cystine uptake by repressing xCT expression and consequently induce ferroptosis in response to ROS-induced stress [27]. Therefore, deficiency of p53 can upregulate xCT expression leading to ferroptosis resistance [27]. However, the p53-mediated regulation of ferroptosis seems to be more complex. Indeed, p53 has been reported to suppress ferroptosis in colorectal cancer cells by blocking the activity of Di-peptidyl-peptidase IV (DDP4) that enhances lipid peroxidation [47]. Moreover, p53 delays the onset of ferroptosis in response to cystine deprivation in a broad range of cell types by upregulating p21 expression which preserves intracellular GSH through a still uncharacterized mechanism [48]. Thus, the ability of p53 to induce or inhibit ferroptosis might be context-dependent.

BAP1 is a nuclear deubiquitinating enzyme involved in the epigenetic regulation of gene transcription [49]. In particular, BAP1 interacts with Additional Sex Combs Like (ASXL) proteins to form the polycomb repressive-deubiquitinase (PR-DUB) complex which removes monoubiquitin from histone 2A (H2A) [49]. Genome-wide analyses revealed that BAP1 epigenetically represses xCT expression leading to lipid peroxidation and ferroptosis induction in a p53-independent manner [4]. Like p53, BAP1 is inactivated in different cancer types. Mutations of BAP1 abolish the ability of cancer cells to promote ferroptosis highlighting the role of epigenetic regulation of ferroptosis in BAP1-mediated tumor suppressive functions [4].

Recently, the CCDC6 has emerged as a new regulator of ferroptotic response [50]. CCDC6 has been characterized as a tumor suppressor gene based on its functions in regulating apoptosis and DNA damage response [51]. Following genotoxic stress, CCDC6 moves into the nucleus and allows DNA repair by maintaining the phosphorylation status of H2A histone family member X (H2AX) required for the assembly of DNA repair proteins at damaged sites. Consequently, loss of CCDC6 impairs homologous recombination repair of DNA double-strand breaks, mimicking a “BRCAness” condition and also determining poly(ADP-ribose) polymerase (PARP)-inhibitors sensitivity in cancer cells [52]. Interestingly, CCDC6 depletion is associated with enhanced xCT expression levels leading to oxidative stress tolerance and ferroptosis resistance in testis germ cells, thus contributing to testicular neoplastic growth through inhibition of ferroptotic cell death [50].

## Role of xCT in multidrug resistance

Despite great advances in the field of cancer therapy, the main challenge for tumor treatment still remains the development of drug resistance. The ability of cancer cells to negatively regulate ferroptosis has been correlated with resistance to anticancer therapies and the induction of ferroptosis has been proposed to overcome chemo-, radio-, targeted- and immune-therapy resistance [53]. Indeed, xCT-mediated ferroptosis inhibition is emerging as a key factor in the development of multidrug resistance making xCT a promising therapeutic target [53].

One of the known mechanisms of cisplatin resistance involves its binding to GSH. Indeed, GSH can bind cisplatin forming cisplatin-GSH adducts which are exported from the cells through the multidrug resistance protein-2 (MRP-2) efflux pump, thus reducing the intracellular levels of active drug [54]. It is well established that cisplatin-resistant cancer cells, including ovarian, lung, and colorectal cancer cells, show increased levels of GSH due to xCT overexpression and that xCT inhibition with sulfasalazine significantly improves sensitivity to cisplatin [55–57].

Temozolomide is the first-line chemotherapy used to treat gliomas, however, acquired resistance is the principal cause of therapy failure [58]. It has been reported that glioma cells exhibit an increased expression of xCT responsible for reduced sensitivity to temozolomide [59]. Moreover, knockdown or pharmacological inhibition of xCT induces GSH depletion, ROS accumulation, and increased glioma cell death under oxidative and genotoxic stress, thereby enhancing their sensitivity to temozolomide [59].

Recently, ferroptosis inducers have been proposed to overcome resistance to epidermal growth factor receptor-tyrosine kinase inhibitors (EGFR-TKIs) [60, 61]. Many epithelial tumors express the adhesion molecule CD44 variant (CD44v), the most prevalent cancer stem cell marker [62]. CD44v regulates the cellular redox status by stabilizing xCT on the plasma membrane and thus promoting the upregulation of GSH synthesis [63]. It has been shown that head and neck squamous cell carcinoma (HNSCC) cells expressing CD44v are intrinsically resistant to EGFR-TKIs due to xCT-dependent cystine transport, whereas differentiated HNSCC cells are CD44v-negative and EGFR-TKIs sensitive [61]. Consistently, xCT inhibitors selectively deplete undifferentiated CD44v-expressing cells and sensitize the undifferentiated CD44v-negative cells to EGFR-TKIs [61].

Radiotherapy is widely used for cancer treatment and involves the use of ionizing radiation to cause massive DNA damage and apoptotic cell death [64]. Recently, it has been demonstrated that radiotherapy is also able to induce ferroptotic cell death and that xCT overexpression abolishes radiation-induced ferroptosis leading to radioresistance [65, 66]. Therefore, xCT inhibitors have been proposed as radiosensitizer agents [65, 67]. In addition, recent studies have demonstrated that immunotherapy and radiotherapy act synergistically to induce tumor-cell ferroptosis through the downregulation of xCT [65]. Cancer immunotherapy aims to restore or enhance the functionality of CD8^+^ cytotoxic T lymphocytes (CTLs), the major killer of cancer cells [68]. It has been reported that immunotherapy-activated CTLs release interferon-gamma (IFN-γ) which suppresses xCT expression in cancer cells promoting ferroptosis [69]. Furthermore, even radiotherapy, independently of immunotherapy, is able to reduce xCT expression in cancer cells through the activation of an ataxia-telangiectasia mutated (ATM)-dependent transcriptional program [65]. These data let us envisage the use of ferroptosis inducers as radiotherapy- and immunotherapy- sensitizers as well as suggest a putative immunotherapeutic/radiotherapeutic combinatorial strategy focused on the xCT modulation.

## xCT limits the metabolic flexibility of cancer cells

During tumor progression, the energy needs of cancer cells undergo a change depending on cell-intrinsic factors, such as the genetic and epigenetic background, but also on cell-extrinsic factors, determined by the microenvironment, such as hypoxia, low nutrient availability, and exposure to anticancer drugs [70]. Generally, cancer cells exhibit a certain degree of metabolic flexibility that allows them to adapt to different nutritional conditions and maintain metabolic fitness [71]. Indeed, metabolic reprogramming is a hallmark of cancer [28].

Recent studies have demonstrated that the increased expression of xCT in cancer cells is necessary to maintain redox homeostasis and suppress ferroptosis, as well as to reduce their metabolic flexibility since cancer cells become highly dependent on glutamine and glucose as a metabolic consequence of xCT-mediated cystine uptake enhancement [1, 72–75]. Glucose and glutamine represent the principal carbon sources for catabolic and anabolic metabolism. Glucose metabolism has a key role in supporting cancer cell survival. Upon transportation into the cell, glucose is phosphorylated by hexokinase to prevent its efflux. Then, glucose is metabolized through glycolysis into pyruvate that is shunted into the tricarboxylic acid (TCA) cycle to produce reducing equivalents nicotinamide adenine diphosphate hydride (NADH) and two chromophores, 1,5-dihydroflavin adenine dinucleotide (FADH_2_) that fuel mitochondrial oxidative phosphorylation [76]. Alternatively, glucose can be processed through the pentose phosphate pathway generating ribose-5-phosphate for nucleic acid synthesis and reducing equivalent NADPH to sustain reductive biosynthetic processes and redox homeostasis [76]. Once transported into cells by xCT, cystine is rapidly reduced through a NADPH-consuming reaction. Therefore, in cancer cells that overexpress xCT the increased cystine influx results in a significant drain on the cytosolic NADPH pool to cystine reduction which makes these cells highly dependent on the pentose phosphate pathway and, thus, on glucose [1]. Several studies have demonstrated that glucose deprivation or inhibition of glucose transporters (GLUTs) in high xCT cancer cells results in intracellular cystine accumulation, the collapse of the redox system due to NADPH depletion, ROS accumulation, and rapid cell death [72, 73, 77]. Conversely, xCT inactivation, by knockdown or pharmacological inhibition, promotes cancer cells’ survival under glucose deprivations [72].

Glutamine is an essential amino acid that participates in many catabolic and anabolic reactions, redox homeostasis, and signal transduction [78]. In particular, glutamine is the major anaplerotic substrate of TCA. Indeed, once imported, glutamine is converted by glutaminase into glutamate which is shunted into the TCA cycle supporting mitochondrial respiration, the major ATP source [78]. Overexpression of xCT increases cystine uptake in exchange for glutamate reducing the intracellular levels of glutamate. Consequently, cancer cells are forced to import more glutamine and activate glutaminase to fuel the TCA cycle and mitochondrial respiration leading to glutamine dependency [1]. An analysis of glutamine uptake and metabolic activities on 46 independently derived breast cell lines, identified a subset of TNBC cells characterized by high xCT expression and cystine consumption rate which are dependent on glutamine uptake [31]. Indeed, pharmacological inhibition of xCT by sulfasalazine attenuates tumor growth in xenograft models [31]. Similarly, cancer cells with hyperactivation of NRF2, which induces xCT expression, or loss of function of Kelch-like ECH-associated protein 1 (KEAP1), a negative regulator of NRF2, show a robust sensitivity to glutamine deprivation or glutaminase inhibition, in part through the increased glutamine export induced by high levels of xCT [74, 75].

In summary, xCT overexpression in cancer cells can induce both glucose dependency, due to the role of glucose in redox maintenance, and glutamine addiction due to glutamate-derived anaplerosis.

## Conclusions

Cancer cells often show higher ROS content compared to normal cells as a consequence of genetic, metabolic, and microenvironment-associated alterations [9]. One of the strategies adopted by cancer cells to survive under conditions of oxidative stress is the upregulation of xCT expression and, thus, the increased cysteine import for GSH biosynthesis to maintain redox homeostasis.

**Figure 1.**
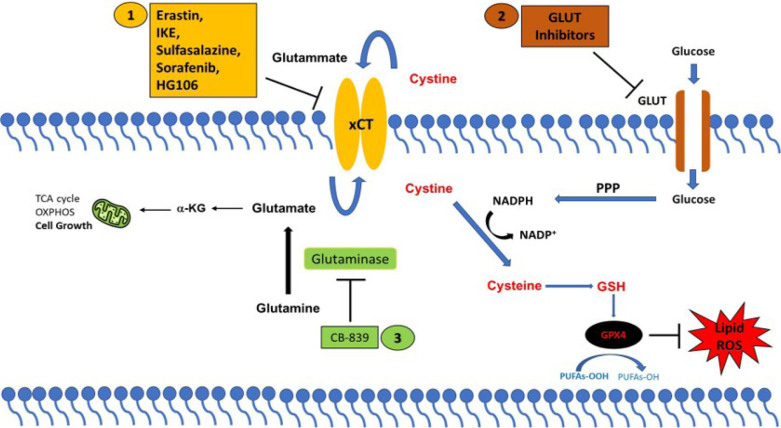
xCT-based therapeutic strategies. Strategies to target cancer cells with high xCT expression. (1) Direct inhibition of xCT; or by targeting xCT-associated metabolic vulnerabilities using (2) GLUT inhibitors and (3) glutaminase inhibitors (CB-839). PPP: pentose phosphate pathway; α-KG: α-ketoglutarate

xCT has been found overexpressed in several tumor types where it regulates many aspects of tumor progression, including cancer cell proliferation and migration, metabolic reprogramming, and drug resistance [79]. The role exerted by xCT in tumor growth is related to its ability to negatively modulate oxidative stress and ferroptotic cell death, recently indicated as a tumor suppressive mechanism [4]. Therefore, xCT has become a fascinating therapeutic target for cancer treatment. The strategies proposed for targeting xCT are summarized in Figure 1.

Different compounds have been identified to directly block the xCT transporter activity and induce ferroptotic cell death, such as sulfasalazine, erastin, imidazole ketone erastin (IKE), and sorafenib [80]. Among these drugs, the most promising is sulfasalazine, a compound with anti-inflammatory properties approved by the Food and Drug Administration (FDA) for the treatment of patient with rheumatoid arthritis. Sulfasalazine is well characterized for selectively suppressing the proliferation of CD44v-expressing cancer stem-like cells and KRAS-mutant cancer cells and markedly inhibiting tumor growth in several pre-clinical cancer models [61]. Erastin has been first identified as an agent that selectively kills cancer cells expressing the small T oncoprotein and oncogenic RAS [81]. To date several studies have demonstrated the anticancer activity of erastin *in vitro*, however, its low solubility and poor metabolic stability prevent its use *in vivo* [80]. Moreover, the erastin analogs piperazine-erastin and IKE, with improved water solubility and metabolic stability, have been shown to induce ferroptosis and limit tumor growth in mouse lymphoma models [82]. Sorafenib is a multikinase inhibitor that has been reported to elicit ferroptosis, regardless of its kinase inhibitor activity, by inhibiting xCT in several cancer cell lines [83, 84]. However, a recent study casts doubt on the sorafenib mechanism of action based on xCT-inhibition mediated ferroptosis, demonstrating that this drug fails to induce ferroptosis across a wide range of cancer cells [85]. Furthermore, the combination of xCT inhibitors with the traditional anticancer therapeutics, chemotherapy, and radiotherapy, but also with targeted therapy and immunotherapy can effectively suppress tumor growth and overcome drug resistance through induction of ferroptosis in numerous cancers [55, 59, 65].

Finally, a further potential therapeutic approach consists in exploiting the metabolic vulnerabilities often associated with xCT overexpression. As mentioned above, cancer cells that upregulate xCT expression are particularly vulnerable to glutamine and glucose deprivation paving the way for the use of glutaminase and GLUT inhibitors in high xCT cancer cells. The glutaminase inhibitor CB-839 has been shown to produce an anti-tumor effect in KRAS-mutant lung cancer cells which carry inactivation of the *KEAP1* gene resulting in NRF2 hyperactivation and xCT overexpression [75, 86]. Moreover, CB-839 suppresses tumor growth in patient-derived xenografts with KEAP1 inactivation with no effects in KEAP1 wild-type tumors [75, 86]. Similarly, the GLUT1/3 inhibitors KL-11743 and BAY-876 selectively induce cell death in xCT-overexpressing cancer cells and inhibit tumor growth in patient-derived xenografts [73].

Overall, the xCT targeting opens to several powerful therapeutic perspectives. However, the unsatisfactory chemical characteristics and off-target effects limit the use of xCT-direct inhibitors in the clinical setting. Therefore, the development of more soluble, stable, and highly specific xCT inhibitors will be critical to expanding therapeutic strategies focused on ferroptosis induction. Likewise, further efforts are needed to identify the cancer types that might most benefit from pro-ferroptotic strategies.

## Abbreviations

BAP1: BRCA1-associated protein 1
CCDC6: coiled-coil domain containing 6
CD44v: CD44 variant
EGFR-TKIs: epidermal growth factor receptor-tyrosine kinase inhibitors
GLUTs: glucose transporters
GPXs: glutathione peroxidases
GSH: glutathione
KEAP1: Kelch-like ECH-associated protein 1
NADPH: nicotinamide adenine dinucleotide phosphate
NOXs: nicotinamide adenine dinucleotide phosphate oxidases
NRF2: nuclear factor E2-related factor 2
RAS: rat sarcoma virus
ROS: reactive oxygen species
TCA: tricarboxylic acid
xCT: solute carrier family 7 member 11

## Declarations

### Copyright

© The Author(s) 2022.
